# Nutrient Protection against Arsenic Toxicity: Folate, Cysteine Support Methylation in Children

**DOI:** 10.1289/ehp.117-a211b

**Published:** 2009-05

**Authors:** Kris Freeman

Nutritional factors are known to influence arsenic metabolism in adults, and poor nutritional status—as reflected in part by a lack of various B vitamins and antioxidants—is thought to confer greater susceptibility to arsenic toxicity. Now researchers working in Bangladesh have reported that deficits in the B vitamin folate and the amino acid cysteine may adversely influence arsenic metabolism in children ***[EHP 117:825–831; Hall et al.]***. The research team also found that, compared with adults, children may metabolize arsenic more efficiently and excrete it more readily, regardless of folate status.

Chronic exposure to arsenic, a known human carcinogen, occurs mainly through contaminated drinking water and currently affects about 140 million people worldwide, including 35 million Bangladeshis. Such exposure has been linked to increased risks of cardiovascular disease and cancers of the skin, bladder, lung, and liver. Childhood exposure also increases the risk of intellectual deficits and respiratory disorders, among other health problems.

Arsenic metabolism involves two methylation steps that rely on folate: Inorganic arsenic (InAs) is first converted to monomethylarsonic acid (MMA), which is then converted to the less toxic dimethylarsinic acid (DMA); this process facilitates urinary arsenic elimination. In the current study, the team measured urinary levels of InAs, MMA, and DMA, as well as blood levels of the metabolic by-product homocysteine and an array of micronutrients (including folate, cysteine, and cobalamin) in 165 6-year-old children. Among the participants, 4.1% of females and 3.3% of males were classified as folate deficient.

Consistent with previous research findings involving adults, higher levels of folate and cysteine correlated with a lower proportion of unmethylated arsenic metabolites in the urine, indicating that adequate levels of these nutrients may be important for arsenic methylation in children. In addition, compared with Bangladeshi adults, children had lower mean proportions of urinary InAs and MMA as well as higher mean proportions of urinary DMA.

The study did turn up a surprise: Plasma homocysteine was inversely correlated with the proportion of MMA in urine, especially in males, but was positively correlated with DMA in urine. Compared with previous findings for adults, the children were also more likely to have high homocysteine levels, despite being less likely to be classified as folate deficient. More research is required to confirm this finding and the underlying mechanisms.

The authors hypothesize that the one-carbon metabolism mechanism behind methylation may be upregulated during periods of rapid growth to meet high demands for DNA and protein biosynthesis. This upregulation would also be associated with an increase in homocysteine biosynthesis. At the same time, behaviors common among Bangladeshi adults—such as cigarette smoking and the chewing of betel nuts—could also play a role in altering arsenic methylation patterns. Overall, this study’s findings indicate that improved nutritional status could constitute a key strategy for reducing the risk of arsenic-related disease in Bangladeshi children.

